# Efficient Distribution of a Novel Zirconium-89 Labeled Anti-cd20 Antibody Following Subcutaneous and Intravenous Administration in Control and Experimental Autoimmune Encephalomyelitis-Variant Mice

**DOI:** 10.3389/fimmu.2019.02437

**Published:** 2019-10-18

**Authors:** Mary-Anne Migotto, Karine Mardon, Jacqueline Orian, Gisbert Weckbecker, Rainer Kneuer, Rajiv Bhalla, David C. Reutens

**Affiliations:** ^1^Centre for Advanced Imaging, The University of Queensland, Brisbane, QLD, Australia; ^2^National Imaging Facility, The University of Queensland, Brisbane, QLD, Australia; ^3^Department of Biochemistry and Genetics, La Trobe Institute for Molecular Science, La Trobe University, Melbourne, VIC, Australia; ^4^Novartis Institutes for BioMedical Research, Novartis Pharma AG, Basel, Switzerland; ^5^ARC Training Centre for Innovation in Biomedical Imaging Technology, Brisbane, QLD, Australia

**Keywords:** radiolabeling, positron emission tomography imaging, monoclonal antibody, neuroimaging, biodistribution, experimental autoimmune encephalomyelitis, subcutaneous, intravenous

## Abstract

**Objective:** To investigate the imaging and biodistribution of a novel zirconium-89 (^89^Zr)-labeled mouse anti-cd20 monoclonal antibody (mAb) in control and experimental autoimmune encephalomyelitis (EAE) mice following subcutaneous (s. c.) and intravenous (i.v.) administration.

**Background:** Anti-cd20-mediated B-cell depletion using mAbs is a promising therapy for multiple sclerosis. Recombinant human myelin oligodendrocyte glycoprotein (rhMOG)-induced EAE involves B-cell-mediated inflammation and demyelination in mice.

**Design/Methods:** C57BL/6J mice (*n* = 39) were EAE-induced using rhMOG. On Day 14 post EAE induction, ^89^Zr-labeled-anti-cd20 mAb was injected in control and EAE mice in the right lower flank (s.c.) or tail vein (i.v.). Positron emission tomography/computed tomography (PET/CT) imaging and gamma counting (*ex vivo*) were performed on Days 1, 3, and 7 to quantify tracer accumulation in the major organs, lymphatics, and central nervous system (CNS). A preliminary study was conducted in healthy mice to elucidate full and early kinetics of the tracer that were subsequently applied in the EAE and control mice study.

**Results:** ^89^Zr-labeled anti-cd20 mAb was effectively absorbed from s.c. and i.v. injection sites and distributed to all major organs in the EAE and control mice. There was a good correlation between *in vivo* PET/CT data and *ex vivo* quantification of biodistribution of the tracer. From gamma counting studies, initial tracer uptake within the lymphatic system was found to be higher in the draining lymph nodes (inguinal or subiliac and sciatic) following s.c. vs. i.v. administration; within the CNS a significantly higher tracer uptake was observed at 24 h in the cerebellum, cerebrum, and thoracic spinal cord (*p* < 0.05 for all) following s.c. vs. i.v. administration.

**Conclusions:** The preclinical data suggest that initial tracer uptake was significantly higher in the draining lymph nodes (subiliac and sciatic) and parts of CNS (the cerebellum and cerebrum) when administered s.c. compared with i.v in EAE mice.

## Introduction

Multiple sclerosis (MS) is an inflammatory, demyelinating autoimmune disease of the central nervous system (CNS) that typically affects the brain and spinal cord (1). Inflammation in early MS pathogenesis is primarily mediated by activated B cells with secondary involvement of T cells (2–8). B cells are produced in the bone marrow, activated in secondary lymphoid organs such as lymph nodes (LNs) and the spleen (5), and play an important role in recognizing and presenting autoantigens to T cells that are involved in MS pathogenesis (4, 9). In addition, presence of B- and T-cell rich tertiary lymphoid structures in the meninges of patients with MS suggest involvement of B- and T-cell interactions that eventually contribute to sustained inflammation in the CNS (10). B cells regulate the activation and differentiation of myeloid antigen-presenting cells and T cells by secretion of distinct pro- and anti-inflammatory cytokines (9, 11). Besides differentiating into autoantibody-producing plasma cells (12), activated B cells express high levels of costimulatory molecules (13) promoting pro-inflammatory differentiation of responding T cells (14), which is likely to contribute directly to development and progression of MS.

CD20 is a surface antigen that is expressed on most B-cell subsets, except pro-B cells, and plasma cells (5, 8). Anti-CD20 therapies selectively deplete CD20+ B cells to reduce inflammation via 3 major mechanisms: complement-dependent cytotoxicity (CDC), antibody-dependent cellular cytotoxicity, and direct cell death pathways (4, 8, 15). Anti-CD20 monoclonal antibodies (mAbs) targeting CD20+ B cells have shown promising results in patients with relapsing-remitting MS (16–18). Use of high-dose intravenous (i.v.) anti-CD20 therapies have shown to achieve maximal long-term B-cell depletion but with slow cellular recovery time (18–20). For mAb-based immunotherapy, subcutaneous (s.c.) administration is preferred over the i.v. route because s.c. route offers unrestricted drainage from the interstitial space allowing mAbs to be absorbed through the lymphatic system (21–23), achieve high localized concentrations in LNs more rapidly (23, 24) and effectively target LNs, where autoreactive B cells interact with autoreactive T cells (2).

Ofatumumab, the first fully human investigational anti-CD20 mAb, has shown potent effector activity (6, 25) at monthly low-dose s.c. administration (26, 27). Ofatumumab binds to a distinct non-continuous CD20 epitope, giving rise to a low off-rate and high avidity resulting in a highly efficient CDC activity. Currently, two Phase 3 trials of ofatumumab are ongoing in patients with relapsing MS (28–30). In preclinical studies, administration of low-dose s.c. vs. high-dose i.v. anti-CD20 therapy showed a similar depletion of CD20+ B cells in circulation and in LNs (26, 31, 32). However, the functional impact of the route of administration (s.c. vs. i.v.) on immune surveillance is not fully elucidated. To understand the relationship between mAb biodistribution as a function of route of administration, a murine experimental autoimmune encephalomyelitis (EAE) model, an induced autoimmune-mediated inflammatory CNS disease and an accepted model of MS was used (33, 34). Using this model, imaging and biodistribution of a novel zirconium-89 (^89^Zr)-labeled mouse anti-CD20 mAb (^89^Zr-labeled anti-CD20 mAb) in the whole body, lymphatic compartments and CNS of EAE and control mice following s.c. and i.v. administration was investigated.

## Materials and Methods

### Experimental Design and Animal Models

Female C57BL/6 mice, aged 12–15 weeks and weighing 21–22 g at baseline, were housed in an animal facility at the Center for Advanced Imaging (Brisbane, Australia) under controlled light (12 h light/dark cycle) and temperature (22–24°C) conditions and provided with food and water as required. Animal experiments were performed using protocols developed at La Trobe University (Melbourne, Australia, AEC#15-90) and translated to the Center for Advanced Imaging with approval from an institutional animal ethics committee (AEC # CAI/233/16). Before the study was performed in the EAE-variant mouse model, a pilot study was conducted in healthy mice to investigate the effect of the route of administration and to elucidate full and early kinetics of the tracer biodistribution following s.c. (*n* = 3–6) and i.v. (*n* = 3–8) injection. The details on experimental design and results for healthy mice are provided in the Supplementary Material. The healthy mice data provided insights to meaningful time points to monitor tracer biodistribution which were subsequently applied in the EAE and control mice study.

**Figure 1:**
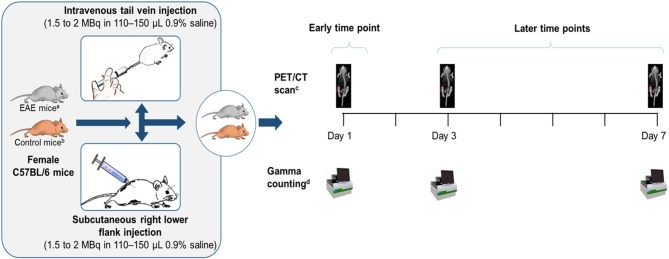
Study design. ^a^C57BL/6 mice post-EAE induction who had reached the peak of the disease on Days 14–15. ^b^Control mice were sham-injected (i.e., subjected to the same procedure as EAE-induced mice, except that rhMOG was replaced with saline). ^c^Whole body clearance and biodistribution of the tracer were assessed by PET/CT imaging. ^d^Organs excised from a subset of mice (*n* = 7–9) and assessed for biodistribution of the tracer by gamma counting. EAE, experimental autoimmune encephalomyelitis; MBq, megaBecquerel; *n*, number of mice; PET/CT, positron emission tomography/computed tomography; rhMOG, recombinant human myelin oligodendrocyte glycoprotein.

On Day 14 post induction, the ^89^Zr-labeled anti-CD20 mAb was administered in EAE and control (sham-injected) mice between 1.5 and 2 MBq in 0.9% saline as either an s.c. right lower flank injection (104–160 μL) or i.v. tail vein injection (110–150 μL) (Figure 1). The injection syringe was filled with approximately 120 μL of the ^89^Zr-labeled anti-CD20 mAb (tracer) and the activity in the syringe was measured using a dose calibrator (CRC-25 PET Radioisotope Dose Calibrator, Capintec Inc., Florham Park, NJ, USA). The activity remaining in the syringe after injection was measured using the same dose calibrator and the total volume injected in each mouse was calculated. Activity concentrations were then expressed as a percent of the decay-corrected injected activity per cm^3^ of tissue, approximated as percentage injected dose per gram (% ID/g).

### EAE Induction

Healthy C57BL/6 mice (15 weeks old) were immunized subcutaneously at the base of the tail with a total of 300 μg recombinant human myelin oligodendrocyte glycoprotein (rhMOG), [extracellular domain (1–125) produced by SinoBiologics in *Escherichia Coli* and was supplied by Novartis Institute for BioMedical Research Switzerland], emulsified in incomplete Freund's adjuvant, supplemented with 4 mg/mL of *Mycobacterium tuberculosis*. The mice received an intra-peritoneal injection of 150 ng of toxic protein from *Bordetella pertussis* in saline at the time of immunization and 48 h later. The control mice were subjected to the same procedure as the EAE-induced mice, except that rhMOG was replaced with saline (sham-injected). EAE induction was performed in a total of 39 EAE mice and 18 control mice. The mice were weighed and examined daily for clinical signs of EAE using standard scoring (0, no paralysis; 1, loss of tail tone; 2, hind limb weakness or paresis; 3, hind limb paralysis; 4, hind limb paralysis and forelimb paresis; 5, moribund or deceased).

### Synthesis and Radiolabeling of the Anti-CD20 mAb

The anti-CD20 antibody was conjugated to p-isothiocyanatobenzyl-desferrioxamine (DFO-NCS) by performing the reaction in a carbonate-bicarbonate buffer (pH 9.2). This provided a simpler way to conjugate the desferrioxamine (DFO) compared with a previous method (35) by avoiding the need to adjust the pH of the reaction mixture. The efficiency of radiolabeling the anti-CD20-antibody-DFO conjugate with ^89^Zr was increased to >90% by continuous shaking and incubating the reaction at 37°C. Use of a spin cartridge further facilitated fast purification and increased the radiochemical concentration, enabling more animals to be screened per production of the tracer. For more details please see Supplementary Material.

### Distribution of the ^89^Zr-Labeled Anti-CD20 mAb

The difference in uptake and biodistribution profiles of the tracer were assessed using positron emission tomography/computed tomography (PET/CT) imaging (Inveon, Siemens, Erlangen Germany) and gamma counting (Wizard 2480 Automated Gamma Counter, Perkin Elmer, Waltham MA, USA) after s.c. and i.v. injections in EAE and control mice on Day 1 (early time point), and Days 3 and 7 (later time points). The whole body clearance of the tracer, expressed as a percentage of the injected dose remaining in the whole body, following s.c. and i.v. injection in control and EAE mice (*n* = 5–9 mice per time point) was assessed. *In vivo* PET/CT imaging was used to assess *in vivo* biodistribution of the tracer following s.c. injection (EAE, *n* = 5–9 mice per time point; control, *n* = 3–6 mice per time point) and i.v. injection (EAE, *n* = 3–4 mice per time point; control, *n* = 1–2 mice per time point). Gamma counting of organs excised from a subset of mice (*n* = 7–9 mice per time point) was used to measure *in vivo* biodistribution of the tracer following s.c. injection (EAE, *n* = 9 mice per time point; control, *n* = 7 mice per time point) and i.v. injection (EAE, *n* = 3–4 mice per time point; control, *n* = 1–3 mice per time point). For gamma counting studies, mice were sacrificed by cervical dislocation and samples of blood and tissues were obtained, weighed, and counted along with a standard solution of the injected dose in a gamma counter. In addition to major organs, tissues collected included LNs from both the upper and lower body (involving both superficial and deep LNs), and CNS compartments (spinal cord regions and brain regions). CNS dissection was carried out on fresh mouse brain, noting that cerebrum (cerebral cortex) contains both gray and white matter (36).

### Statistical Analysis

The PET sinograms were reconstructed with FBP (filtered back-projection) and an ordered-subset expectation maximization (OSEM2D) algorithm was then analyzed using the Inveon Research Workplace Software (IRW 4.1, Siemens, Erlangen, Germany). This allows fusion of CT and PET images and definition of regions of interest (ROI). Three-dimensional ROI were placed within the whole body, as well as all the organs of interest such as the liver, spleen, and kidneys, using morphologic CT information to delineate organs. In biodistribution and imaging studies, the correlation between tracer uptake in the lymphatic tissue between the groups (EAE and control mice) and within the groups was assessed using single factor analysis of variance (ANOVA), two-factor ANOVA and one-way ANOVA. *P*-values less than alpha (α = 0.05) were considered significant. CNS biodistribution in EAE and control mice was compared using a χ squared test of independence to examine the relationship between tracer uptake within the CNS and EAE disease state. χ^2^ values greater than $\chi_{\text{crit}}^{2}$ = 3.84 and *p*-values less than alpha (α = 0.05) were considered significant. Comparisons between the s.c. and i.v. routes of administration of the tracer and its subsequent biodistribution and accumulation in different organs, lymphatics and CNS was analyzed using a Student's *t*-test. All data are expressed as mean ± standard error of the mean (SEM), unless otherwise specified.

## Results

### Clinical Profiling of the EAE Model: Clinical Onset and Peak of the Disease

**Figure 2:**
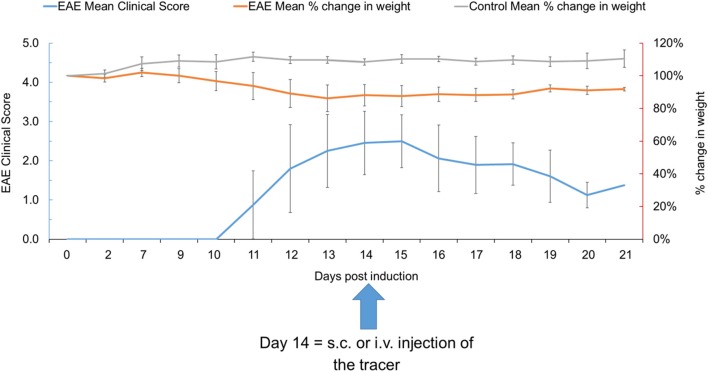
The EAE mean clinical score and percent change in weight in EAE and control mice at various time points (*n* = 39). Data presented as mean ± SD. EAE, experimental autoimmune encephalomyelitis; i.v., intravenous; s.c., subcutaneous; SD, standard deviation.

EAE-induced mice experienced weight loss 9–10 days after EAE induction and ambulatory difficulties started to appear from 10 to 12 days post induction, with low to moderately severe clinical scores (0.5–3.0) and 100% disease incidence. The peak of the disease was observed at 13–15 days post induction, with mean clinical scores of 2.5 ± 0.6 on 14–15 days post induction, which gradually dropped to 1.7 ± 0.3 at 21 days post induction. The mean percent change in body weight was 18% at 17 days post induction which was in line with the clinical scores observed (Figure 2). Control mice did not experience weight loss or any clinical score throughout the experiment.

### Biodistribution of the ^89^Zr-Labeled Anti-CD20 mAb in EAE and Control Mice Following S.C. and I.V. Administration

#### Whole Body Clearance

**Figure 3:**
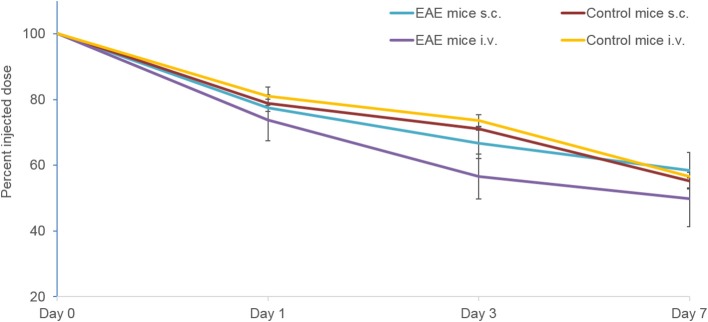
Whole body clearance of the ^89^Zr-labeled anti-CD20 mAb following s.c. and i.v. injection in control and EAE mice (*n* = 5–9 mice per time point) measured using PET/CT. Data presented as mean ± SEM. i.v., intravenous; mAb, monoclonal antibody; n, number of mice; s.c., subcutaneous; SEM, standard error of the mean ^89^Zr, Zirconium-89.

In EAE mice, the proportion of the tracer remaining in the whole body at Day 7 following s.c. injection (58.5 ± 5.4%) was comparable to that observed following i.v. injection (49.9 ± 8.6%) (Figure 3). A similar trend was observed in control mice with the proportion of the tracer remaining in the whole body following s.c. and i.v. injection being similar at Day 7 (55.3 ± 2.5% and 56.5 ± 0.3%, respectively).

#### PET/CT Imaging

**Figure 4:**
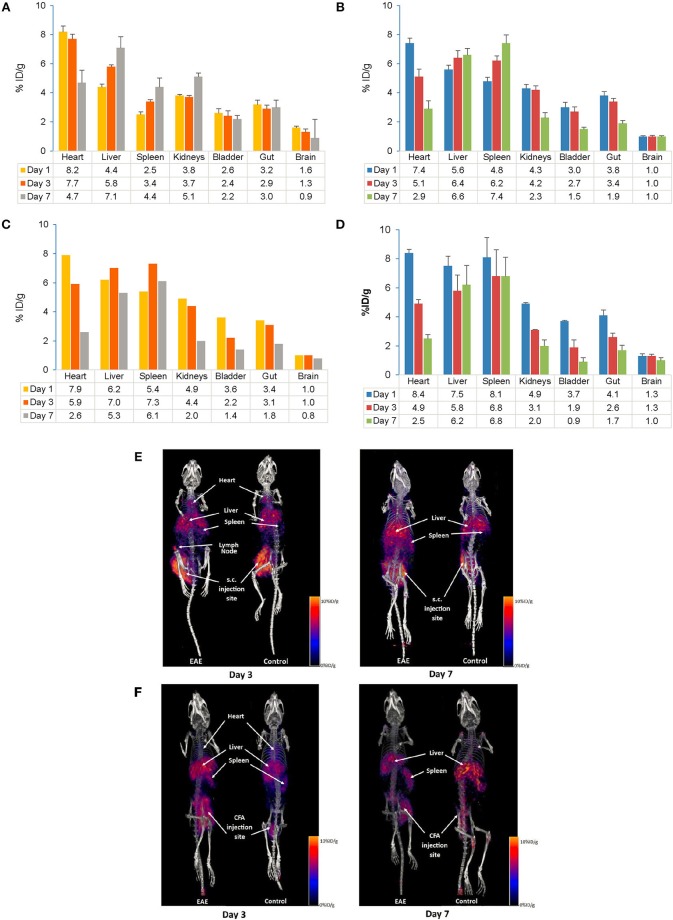
Comparison of PET/CT *in vivo* biodistribution **(A–D)** and *in vivo* imaging **(E,F)** of the ^89^Zr-labeled anti-CD20-mAb in control and EAE mice following s.c. and i.v. injection. **(A)** Biodistribution of the tracer following s.c. injection in control mice (*n* = 3–6). **(B)** Biodistribution of the tracer following s.c. injection in EAE mice (*n* = 3–9). **(C)** Biodistribution of the tracer following i.v. injection in control mice (*n* = 1–2)^#^. **(D)** Biodistribution of the tracer following i.v. injection in EAE mice (*n* = 3–4). **(E)** *In vivo* imaging following s.c. injection of the tracer in control and EAE mice. **(F)** *In vivo* imaging following i.v. injection of the tracer in control and EAE mice. Data presented as mean ± SEM. ^#^Sample size was very low (*n* = 1 or 2) to calculate the SEM values. % ID/g, percentage injected dose per gram; EAE, Experimental Autoimmune Encephalomyelitis; i.v., intravenous; mAb, monoclonal antibody; *n*, number of mice; PET/CT, positron emission tomography/computed tomography; s.c., subcutaneous; SEM, standard error of the mean; ^89^Zr, Zirconium-89.

The distribution of the tracer following s.c. and i.v. injection was measured on Days 1, 3, and 7. In control mice, tracer uptake following s.c. injection at Day 7 was highest in the liver followed by the kidney (Figure 4A), while in EAE mice it was highest in the spleen and liver (Figure 4B). Tracer uptake in the remaining peripheral organs (heart, kidneys, bladder, and gut) on Day 7 was slightly higher in the control mice compared with the EAE mice. Following i.v. injection, tracer accumulation on Day 7 was highest in the spleen and liver in control (Figure 4C) and EAE mice (Figure 4D). In both EAE and control mice, the initial exposure (Day 1) of the tracer following i.v. injection was highest in the heart, spleen and liver and gradually decreased by Day 7. Composite PET/CT images of control and EAE mice following s.c. and i.v. injections are shown in Figures 4E,F.

#### Gamma Counting

##### Overall distribution

**Figure 5:**
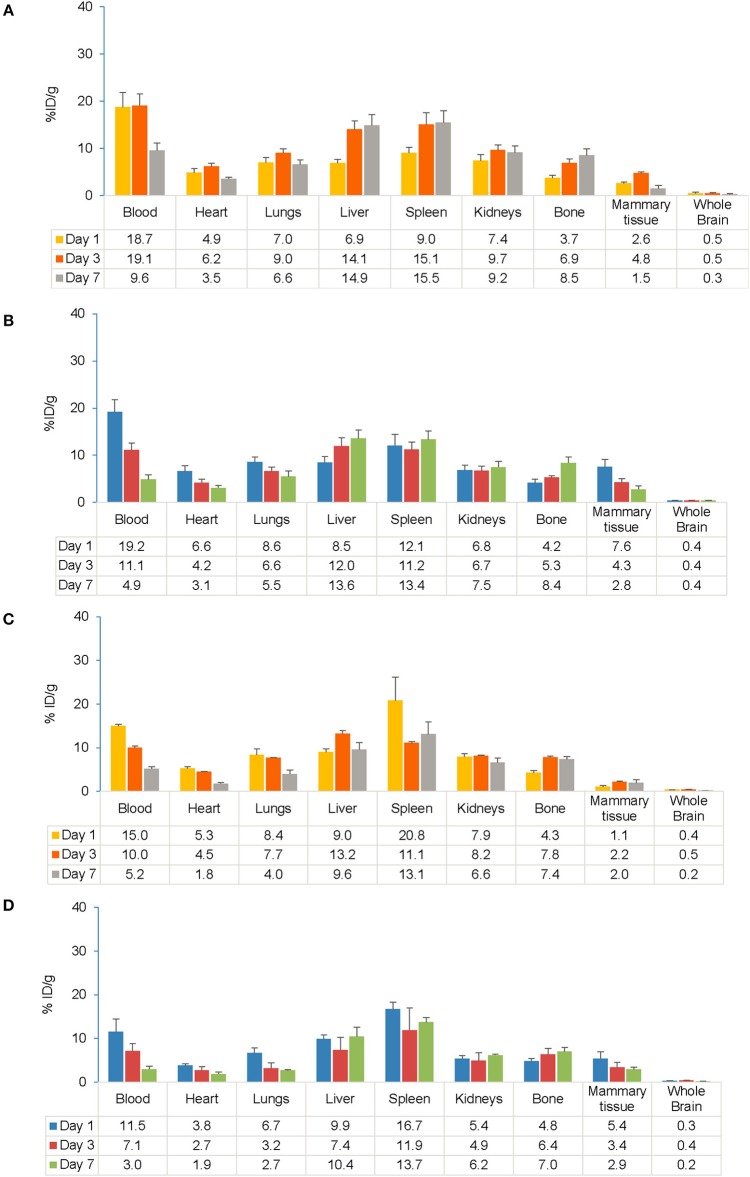
Comparison of gamma counter biodistribution of the ^89^Zr-labeled anti-CD20 mAb in control and EAE mice following s.c. **(A,B)** and i.v. **(C,D)** injection. **(A)** Biodistribution of the tracer following s.c. injection in control mice (*n* = 7). **(B)** Biodistribution of the tracer following s.c. injection in EAE mice (*n* = 9). **(C)** Biodistribution of the tracer following i.v. injection in control mice (*n* = 1–3). **(D)** Biodistribution of the tracer following i.v. injection in EAE mice (*n* = 3–4). Data presented as mean ± SEM. % ID/g, percentage injected dose per gram; EAE, experimental autoimmune encephalomyelitis; i.v., intravenous; LNs, lymph node; mAb, monoclonal antibody; *n*, number of mice; s.c., subcutaneous; SEM, standard error of the mean; ^89^Zr, Zirconium-89.

Gamma counting of organs from a subset of mice was conducted on Days 1, 3, and 7 after injection of the tracer. Following s.c. injection, higher accumulation of the tracer was observed in blood, the spleen and liver, which was consistent between EAE and control mice up to Day 7 (Figures 5A,B). Following i.v. injection, a high tracer accumulation was observed in the spleen, blood, and liver on Day 1 that was sustained up to Day 7 in the spleen and liver in both EAE and control mice (Figures 5C,D). Tracer uptake in peripheral tissues and all major organs was similar between EAE and control mice, except for mammary tissue, where the initial tracer exposure was higher in EAE mice than controls. Gamma counting analysis in the EAE model showed high tracer accumulation in the spleen following s.c. and i.v. injection, respectively. Comparing s.c. with i.v. routes of administration, the accumulation of the tracer in all the major organs including the spleen, kidneys, lungs, heart, liver, blood, and mammary tissue showed no significant difference.

##### Lymphatic tissue

**Figure 6:**
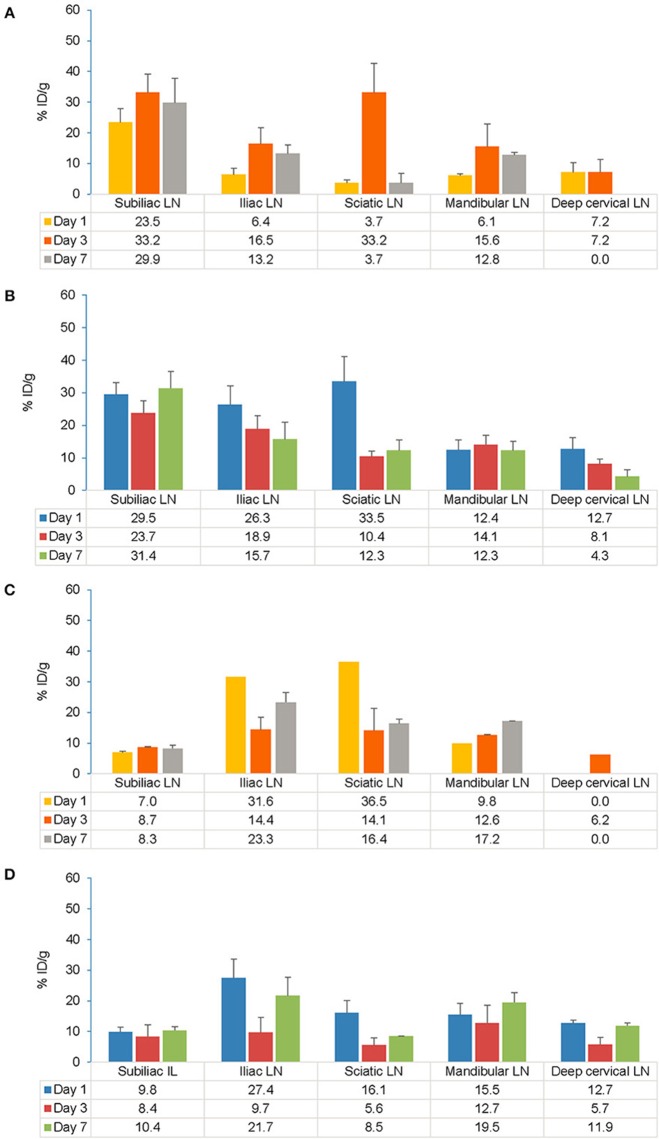
Comparison of gamma counter biodistribution of the ^89^Zr-labeled anti-CD20 mAb in control and EAE mice across specific LNs following s.c. **(A,B)** and i.v. **(C,D)** injection. **(A)** Biodistribution of the tracer in specific LNs following s.c. injection in control mice (*n* = 7). **(B)** Biodistribution of the tracer in specific LNs following s.c. injection in EAE mice (*n* = 9). **(C)** Biodistribution of the tracer in specific LNs following i.v. injection in control mice (*n* = 1–2)^#^. **(D)** Biodistribution of the tracer in specific LNs following i.v. injection in EAE mice (*n* = 3–4). Data presented as mean ± SEM. ^#^Sample size was very low (*n* = 1 or 2) to calculate the SEM values for Iliac LN, Sciatic LN, mandibular LN on Day 1 and for deep cervical LN on Day 3 following i.v. injection in control mice. A 2-way analysis of variance (ANOVA) test was applied to detect significant association between tracer uptake and EAE. % ID/g, percentage injected dose per gram; EAE, experimental autoimmune encephalomyelitis; i.v., intravenous; LLOD, lower limit of detection (less than 3x background signal); LNs, lymph nodes; mAb, monoclonal antibody; n, number of mice; s.c., subcutaneous; SEM, standard error of the mean; ^89^Zr, Zirconium-89.

Following s.c. injection, the highest accumulation of the tracer was observed on Day 7 in the subiliac LN (also known as inguinal LN) that drains the s.c. injection site in both EAE and control mice **(**Figures 6A,B). Across all the time points in EAE mice, the ranges of tracer uptake in the subiliac LN was 11–59% ID/g (vs. 10–65% ID/g in control), 7–51% ID/g (vs. 6–33% ID/g in control) in the iliac LN, 6–39% ID/g (vs. 2–46 % ID/g in control) in the sciatic LN and 4–26% ID/g (vs. 5–30% ID/g in control) in the mandibular LN. No significant association was observed between LN tracer uptake in EAE vs. control mice after s.c. administration as assessed by ANOVA. For each LN and across all time points, both within and between EAE and control mice, the F values were consistently greater than F_crit_ (4.60, the lowest value) and the *p*-values were greater than alpha (0.05), indicating no difference between EAE and control mice after s.c. administration.

Following i.v. injection, the tracer uptake in both control and EAE mice on Day 7 was highest in the iliac and sciatic LNs, closest to the i.v. injection site (Figures 6C,D). Across all the time points in EAE mice, the ranges of tracer uptake in iliac LN was 7–38% ID/g (vs. 8–31% ID/g in control), sciatic LN was 2–29% ID/g (vs. 4–26% ID/g in control), mandibular LN was 6–17% ID/g (vs. 9–17% ID/g in control), subiliac LN was 4–16% ID/g (vs. 4–9% ID/g in control) and deep cervical LN was 0–14% ID/g (vs. 0–6% ID/g in control). Analysis of variance revealed no significant association between tracer uptake and EAE mice. Following i.v. injection, the tracer accumulation across all time points for each LN as presented by the F values were consistently greater than *F*_crit__(5.31)_ and *p*-values were greater than alpha (0.05) indicating no difference between EAE and control mice except for in the deep cervical LN which was significant in EAE vs. control mice (*p* < 0.01 and *F* = 14.45). When comparing s.c. and i.v. routes, significant uptake of the tracer was observed on Day 1 in the draining LNs i.e., in subiliac LN following s.c. injection (*t*-test, *p* = 0.0067). However, this difference was no longer significant by Days 3 and 7.

##### Central nervous system

**Figure 7:**
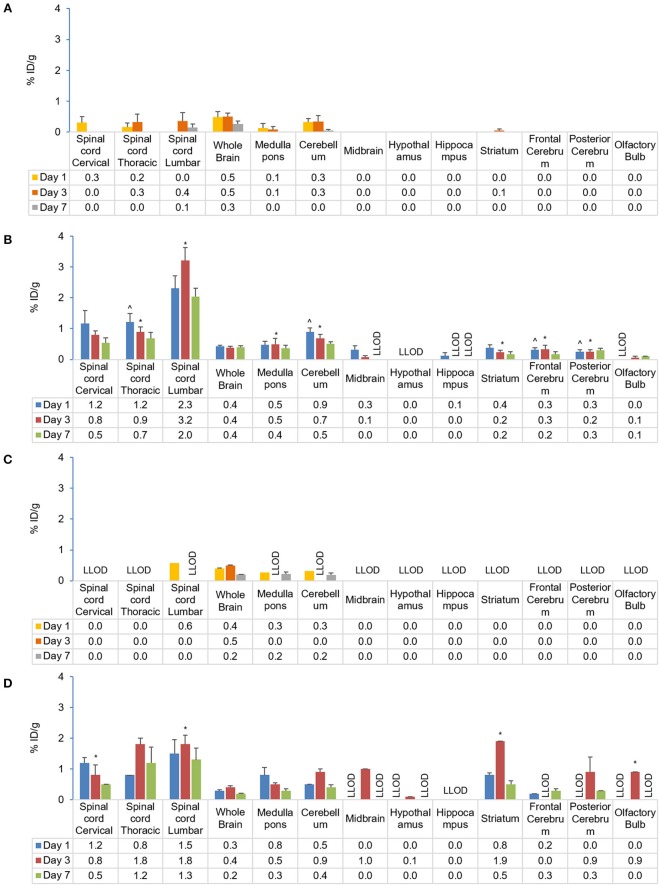
Comparison of gamma counter biodistribution of the ^89^Zr-labeled anti-CD20 mAb in control and EAE mice across CNS following s.c. **(A,B)** and i.v. **(C,D)** injection. **(A)** Biodistribution of the tracer in the CNS following s.c. injection in control mice (*n* = 4). **(B)** Biodistribution of the tracer in the CNS following s.c. injection in EAE mice (*n* = 9). **(C)** Biodistribution of the tracer in the CNS following i.v. injection in control mice (*n* = 1–2)^#^. **(D)** Biodistribution of the tracer in CNS following i.v. injection in EAE mice (*n* = 4–5). *p < 0.05, using a Chi-squared test of independence (for s.c.: χ^2^ [1, *N* = 39] = 6.6–22.5, α = 0.05; i.v.: c^2^ [1, *N* = 15] = 5.0–16.16, *p* < 0.05) to compare CNS biodistribution of the tracer in control and EAE mice, highlighting significant differences in the relationship between CD20 antibody uptake and EAE. ^∧^*p* < 0.05, using single factor ANOVA [*F*_(1)_ > 4.84; *p* < 0.05] and independent *t*-test to compare CD20 antibody uptake in EAE mice following s.c. and i.v. administration, suggesting regional early preferential CNS uptake in EAE following s.c. administration. Data presented as mean ± SEM. ^#^Sample size was too low (*n* = 1 or 2) to calculate the SEM values for lumbar spinal cord, medulla/pons, and cerebellum on Day 1 following i.v. injection in control mice. % ID/g, percentage injected dose per gram; Ab, antibody; CNS, central nervous system; EAE, experimental autoimmune encephalomyelitis; i.v., intravenous; LLOD, lower Limit of detection (less than 3x background signal); mAb, monoclonal antibody; N, total number of mice; n, number of mice; s.c., subcutaneous; SEM, standard error of the mean; ^89^Zr, Zirconium-89.

In EAE mice, high initial tracer uptake following s.c. injection was observed in the lumbar spinal cord with 2.3% ID/g (vs. 0.0% ID/g in control) that increased to 3.2% ID/g on Day 3 and fell to 2.0% ID/g on Day 7 (Figures 7A,B). The relationship between tracer uptake and EAE, analyzed using a χ squared test of independence, was found to be significant for the spinal cord (cervical, thoracic, lumbar; *p* < 0.001 for all), cerebellum (*p* < 0.001), medulla' pons (*p* = 0.002), striatum (*p* = 0.009), and cerebrum (frontal and posterior areas; *p* < 0.001 for both) (χ^2^ [1, *n* = 39] = 6.6–22.5; *p* < 0.05). Further analysis to test the relationship between tracer uptake and EAE disease state (i.e., mice with moderate and mild EAE symptoms) did not show a statistically relevant difference between the 2 groups. Following i.v. injection, EAE mice showed higher tracer uptake on Day 7 in the spinal cord (lumbar, 1.3% ID/g; thoracic, 1.2% ID/g; cervical, 0.5% ID/g) vs. controls (0% ID/g for all) (Figures 7C,D). The relationship between tracer uptake and EAE, analyzed using a χ squared test of independence, was significant for the lumbar (*p* = 0.025) and cervical spinal cord (*p* = 0.003), striatum (*p* = 0.025) and olfactory bulb (*p* < 0.001) (χ^2^ [1, *n* = 15] = 5.0–16.16, *p* < 0.05). The relationship between tracer uptake within the CNS and EAE disease states (i.e., in mice with high and low clinical scores) was not observed. Within the CNS, significantly higher uptake of the tracer was observed in the thoracic spinal cord (*p* = 0.0455), cerebellum (*p* = 0.0333), and frontal cerebrum (*p* = 0.0237) of the brain on Day 1 following s.c. compared with i.v. administration. However, by Days 3 and 7, these differences were no longer significant between s.c and i.v. administration. A comparison of tracer uptake in EAE across all time points for different routes of administration was significant only for the cerebellum (*p* = 0.017) and frontal (*p* = 0.026) and posterior cerebrum (*p* < 0.001) (Chi-squared test of independence; χ2 [1, *n* = 37] = 5.0–12.1, *p* < 0.05).

## Discussion

The present study investigated differential uptake and biodistribution profiles of the ^89^Zr-labeled anti-CD20 antibody following s.c. and i.v. administration in EAE, control and healthy mice (Supplementary Material) using PET/CT imaging and gamma counting. From PET/CT data, the proportion of tracer remaining in the whole body at Day 7 following s.c. injection (58.5 ± 5.4%) was found comparable to the proportion remaining following i.v. injection (49.9 ± 8.6%). Irrespective of the route of administration, the PET/CT imaging data showed strong evidence of biodistribution of tracer in all the major organs, which was further validated by *ex vivo* gamma scintillation of tissues. *Ex vivo* tissue biodistribution analysis following both s.c. and i.v. injections of the tracer in EAE mice showed similar patterns of tracer uptake in major organs when compared with control (sham-injected) and healthy mice. Overall biodistribution of tracer in the lymphoid organs and CNS of EAE mice was consistent with both routes of administration, except for draining LNs and parts of the CNS including the spinal cord, cerebellum and cerebrum that were significantly higher for the s.c. vs. i.v. route of administration at early time points.

The lymphatic system plays a crucial role linking the peripheral immune system and the CNS (37). Evidence suggests that CNS lymphatics could possibly provide a route for B-cell trafficking that bypass the peripheral circulation, allowing for continued B-cell maturation in LNs (37–39). Lymphatics represent the primary absorptive pathway for macromolecules such as proteins (>16 kDa molecular mass). Preclinical studies suggest that LN exposure is proportionally related to the molecular weight of the protein and dose absorbed by the lymphatic system (22, 23). Therefore, mAb-based therapies administered through the s.c route can target B-cell-rich LNs and can be a potential therapeutic option to treat autoimmune diseases such as MS. Gamma counting analysis of s.c. EAE mice at early time points showed higher levels of tracer accumulation in blood (19.2% ID/g) followed by the spleen (12.1% ID/g), in contrast i.v. injection showed higher tracer accumulation in the spleen (16.7% ID/g) followed by blood (11.5% ID/g). The results with s.c. administration were consistent with earlier findings that showed mAbs administered subcutaneously enter the interstitium and follow a decreasing pressure gradient toward the lymphatics subsequently reaching draining LNs (40) where autoreactive B cells prime T cells resulting in their activation. In our study, s.c. administration resulted in high tracer uptake in the superficial subiliac LNs and deep sciatic LNs compared with the i.v. administration. Tracer distribution in the deep iliac LNs in the lower body, superficial mandibular LN, and deep cervical LNs in the upper body were comparable between s.c. and i.v. administrations. Similar findings were observed in previous studies, where s.c. dosing achieves high localized concentrations of mAbs in LNs more rapidly than i.v. dosing (22, 23). Specific access to the lymphatics after s.c. administration has potential utility in targeting delivery of mAbs directly to the innate immune system and has considerable therapeutic and pharmacological ramifications.

Cells of the B-cell lineage have been found to persist in the CNS of MS patients and occupy multiple sub-compartments, including the cerebrospinal fluid, parenchymal white matter lesions, and meninges (37). Evidence from somatic mutation analysis has demonstrated that the same B-cell clones may occupy all three CNS sub-compartments, raising the central question as to exactly how and where such clones initially access the CNS and how they communicate across these CNS sub-compartments (41). Another major unanswered question is the timing of the first entry of B cells in the CNS during MS disease pathophysiology, which could be addressed following confirmation of the definitive correlation of this CD20 PET signal with the presence of B cells in the CNS. By injecting the tracer early in the disease course, it might be possible to determine the timing of the earliest evidence of the CD20 B-cell subset in the CNS and the relationship of this timing with clinical onset (42), but further experiments would be needed to prove B-cell specificity of our tracer in this animal model, example, repeating the experiment in the CD-20 deficient mice, using isotype negative control mAb or by doing blocking studies with cold antibody. Additionally, it may be possible to determine the differential effects of anti-CD20 targeting, if any, on the CNS and lymphoid tissues over the disease trajectory.

This study has, demonstrated an increased uptake of tracer in the CNS of EAE mice compared with control mice for the first time following either s.c. or i.v. administration. Across all time points, tracer uptake was significantly higher for s.c. compared to i.v. administration in EAE mice in the cerebellum and frontal and posterior cerebrum and at early time points this difference was also observed in the thoracic spinal cord, suggesting early preferential CNS uptake following s.c. administration. Cerebellar cortex is a major predilection site for demyelination and the cortex region is affected independently of white matter lesions in MS (43). Pathological studies have revealed that in the standard EAE rodent model of MS, the cortex remain largely unaffected (44). Interestingly, in our study, a significant uptake of the tracer in the cerebrum (cortical regions containing both gray and white matter) was observed following either s.c. or i.v. administration. This could be explained by the fact that in the standard EAE model, the disease process is T-cell driven, potentially resulting in differences in lesion composition and topography (45). Additionally, it may be of clinical relevance that cortical inflammation is a transient phenomenon, and that cortical demyelination is readily compensated by rapid remyelination in a rat model (44). With chronicity, diffuse inflammation accumulates throughout the whole brain and is associated with progressive axonal injury and cortical demyelination leading to cerebellar dysfunction in MS (46). In this study, the results highlight that with s.c. administration, tracer could reach certain parts of CNS more rapidly and may be efficient at delivering antibodies to the CNS when compared with the i.v. administration in EAE mice. However, its clinical significance is uncertain at this stage. Accumulation of the tracer within multiple CNS regions, including the spinal cord, striatum, pons, medulla, hippocampus, and cerebrum, suggest that by the peak of the disease, a CD20 positive population might be established in these regions of the CNS which are associated with symptoms commonly identified and regions targeted in MS (47).

Using a spatio-temporal mapping method of EAE disease (48) in C57BL/6 mice to identify reproducible lesion topography along the whole of the neuraxis and associated B- and T-cell infiltration, this study further demonstrated rhMOG-induced EAE as a successful model of CNS infiltration of B cells. The results provide additional evidence that neuroinflammation in the spinal cord and brain of EAE-induced mice could be detected using this ^89^Zr-labeled anti-CD20-mAb. This may potentially offer a sensitive technique for detecting and monitoring neuroinflammatory lesions and B-cell uptake in the CNS, particularly the spinal cord of MS patients. The biodistribution results revealed higher tracer uptake in the brain and spinal cord of EAE mice in this model of MS. However, further studies are required to confirm the B-cell specificity of radiolabeled tracers in MS patients to translate into a practical tool for detecting and monitoring B cells in disease progression and treatment.

The current data found no correlation between maximum clinical scores and CNS tracer uptake during EAE progression, confirming the degree of heterogeneity in immunological responses. Caravagna et al. recently highlighted the complex *in vivo* recruitment of innate immune cells in EAE in multiplex patterns (49); however, due to the broad analysis conducted in the present study, such complex changes through disease progression would not be accurately tracked.

In conclusion, the ^89^Zr-labeled anti-CD20 mAb demonstrated effective absorption from the injection site and biodistribution to all major organs was consistent with both routes of administration in EAE and control mice. PET/CT imaging confirmed rapid and specific localization of the tracer to the B-cell compartment. A good correlation was observed between *in vivo* PET/CT data and the *ex vivo* quantification of biodistribution of the tracer. Initial tracer uptake within the lymphatics was found to be higher in the draining LNs, with highest uptake in the subiliac and sciatic LN following s.c. administration compared with i.v. administration as measured by gamma counting in EAE mice. The tracer uptake within the CNS was clearly higher in EAE vs. control mice. Moreover, the uptake in the lumbar spinal cord was higher following s.c. vs. i.v administration. The cerebellum and cerebrum counts at 24 h suggest after s.c. administration tracer reached certain parts of CNS more rapidly and may be more efficiently target these sites within the CNS when compared with i.v. administration. The biodistribution studies also suggests that the rhMOG-induced EAE-variant mouse model successfully illustrates CNS infiltration of B cells and that neuroinflammation in the spinal cord and brain of EAE-induced mice could be detected using a fully validated ^89^Zr-labeled anti-CD20 mAb.

## Data Availability Statement

All datasets generated for this study are included in the manuscript/Supplementary Files.

## Ethics Statement

The animal study was reviewed and approved by La Trobe University (Melbourne, Australia, AEC#15-90) and Centre for Advanced Imaging institutional animal ethics committee (AEC # CAI/233/16).

## Author Contributions

DR was involved in the study concept, experiment design, and contributed to interpretation of results and development of the manuscript. M-AM performed experiments, analyzed data, and contributed to interpretation of results and manuscript development. RB was involved in experimental design, planned studies, performed experiments, contributed to data analysis and interpretation, and also manuscript development. KM performed experiments, contributed to data analysis and interpretation, and development of the manuscript. JO provided training in the generation of EAE and contributed to interpretation of results and manuscript development. GW and RK contributed to study concept, data interpretation, and manuscript development.

### Conflict of Interest

The authors declare that this study received funding from Novartis Pharma AG. GW and RK (funders from Novartis Pharma AG) contributed to study concept, data interpretation, and manuscript development. GW and RK are employees of Novartis. The institution (The University of Queensland) of M-AM, RB, KM, and DR received research support from Novartis. JO received research support from Novartis.
