# Supplementary material for: Efficient Distribution of a Novel Zirconium-89 Labeled Anti-cd20 Antibody Following Subcutaneous and Intravenous Administration in Control and Experimental Autoimmune Encephalomyelitis-Variant Mice
Source: Front Immunol. 2019 Oct 18;10:2437. doi: 10.3389/fimmu.2019.02437 (PMC6813232; doi:10.3389/fimmu.2019.02437)
Supplement: Supplementary file 1 [file Data_Sheet_1.PDF]

Supplementary Material

1 Synthesis of the anti-CD20 antibody-DFO antibody conjugate

In a typical procedure, 0.2 mL of anti-CD20-monoclonal antibody (approx. 2mg of anti-CD20 mAb) was added to an Eppendorf vial containing 0.8 mL of a carbonate-bicarbonate buffer (pH 9.2) to give a 2 mg/mL solution. Approximately 50 nmol of p-isothiocyanatobenzyl desferrioxamine (DFO-NCS) in approximately 5 µL of dimethyl sulfoxide (DMSO) was added to the Eppendorf tube and the suspension was incubated at 37°C at 400 rpm for 1 hour using a Thermomixer. After 1 hour, the resulting anti-CD20 mAb-DFO was purified using an Amicon Ultra 0.5mL centrifugal filter (30kDa cut-off). The purified anti-CD20 mAb-DFO was formulated in 1 mL of a 0.5M HEPES buffer (pH 7.5)

2 Radiosynthesis of the ^{89}Zr-labeled anti-CD20 mAb

Depending on the activity concentration, 20 to 100 µL of Zr-89 oxalate (30 to 45 MBq) was transferred to an Eppendorf vial. If the total volume of Zr-89 oxalate was less than 100 µL, the volume was the adjusted to 100 µL with the addition of 1 M oxalic acid. To this, 110 µL of 1 M sodium carbonate was added and the contents in the vial were swirled resulting in a Zr-89 solution with a pH of approximately 7. This solution was then transferred to another Eppendorf vial containing the 400 µg of anti-CD20 antibody-DFO in 200 µL 0.5 M HEPES buffer. Using the Thermomixer, this vial was incubated at 37°C at 400 rpm for 1 hour. After 1 hour, the resulting ^{89}Zr-labeled anti-CD20 mAb-DFO conjugate was purified using an Amicon Ultra 0.5mL centrifugal filter (30 kDa cut-off). The purified ^{89}Zr-labelled anti-CD20 mAb-DFO conjugate was formulated using 600 µL of 0.9% saline (pH 6.5). Purity was assessed using a iTLC strip (part number SGI0001, purchased from Agilent technologies) and 50 mM DTPA solution (adjusted to pH 5.5 with 1 M NaOH). Under these condition, ^{89}Zr-labelled anti-CD20 mAb-DFO conjugate stays on the baseline and free ^{89}Zr moves to the solvent front. After purification, the compound was found to be stable with radiochemical purity of 100% which was further confirmed after 24 hours (**Supplementary Figure 1**).

3 Biodistribution of the ^{89}Zr-labeled anti-CD20 mAb in healthy mice

The ^{89}Zr-labeled anti-CD20 mAb was injected into C57BL/6 healthy mice (approximately 40 µg/mouse) either as an i.v. tail vein injection (~2.3 MBq/mouse) or s.c. right lower flank injection (~2.2 MBq/mouse) (**Supplementary Figure 2**). Whole body clearance and biodistribution of the ^{89}Zr-labeled anti-CD20 mAb was monitored by positron emission tomography/computed tomography (PET/CT) imaging at 4 hours and Days 1, 2, 3 and 7. From a subset of mice (n=3−8), organs were excised and gamma counting was performed on Days 1, 3 and 7 post injection to study the biodistribution of the ^{89}Zr-labeled anti-CD20 mAb.

3.1 Whole body clearance

The proportion of the ^{89}Zr- labeled anti-CD20 mAb remaining in the whole body at Day 7 following i.v. injection (62.9±5.04%) was comparable to that observed following s.c. injection (55.3±4.31%) (**Supplementary Figure 3**)

3.2 Biodistribution of the ^{89}Zr-labeled anti-CD20 antibody by PET/CT imaging

Following i.v. injection of the tracer, a high uptake of the ^{89}Zr-labeled anti-CD20 mAb was observed in the liver with values of 5.5% ID/g at Day 7 (**Supplementary Figure 4A**). The uptake of the tracer in the spleen showed a trend similar to the liver, with a value of 6.8% ID/g at Day 1 which fell to 5.0% ID/g at Day 3, and increased to 6.6% ID/g by Day 7. Higher levels of the tracer in all of the major organs at early time points are likely to be indicative of high levels of circulating blood carrying the tracer.

Following s.c. injection of the tracer, the highest uptake of the tracer was observed in the heart and liver with values above 4% ID/g from Day 1 after injection (**Supplementary Figure 4B**). In the spleen, the tracer uptake was found to be 2.5% ID/g on Day 1, increasing to 3.4% ID/g on Day 3 and falling to 4.4% ID/g on Day 7. The distribution pattern following s.c. injection closely followed the pattern seen with i.v. injection (Figure 4). Following s.c. injection, a 24 hour lag with significant levels of the tracer not entering the blood stream was observed compared with i.v. injection. The initial spleen exposure to the tracer was much higher after i.v. administration than after s.c. administration. Composite PET/CT images of mice following i.v. and s.c. injections of the ^{89}Zr-labeled anti-CD20 mAb are shown in **Supplementary Figure 4C** and **4D.**

3.3 Biodistribution of the ^{89}Zr-labeled anti-CD20 antibody by gamma counting

Following i.v. injection, a high accumulation of the ^{89}Zr-labeled anti-CD20 mAb was observed in the spleen, lungs, liver, kidneys and inguinal lymph nodes (LNs) (**Supplementary Figure 5A**). At an early time point (Day 1), high levels of activity were observed in blood (17.0% ID/g) and in highly perfused organs. At a later time point (Day 7), the highest retention of the tracer was observed in the spleen (24.9% ID/g), liver (10.2% ID/g), LNs (8.3% ID/g) and kidneys (8.1% ID/g).

Following s.c. injection, an initial high accumulation of the tracer was seen in blood, LNs, the spleen, kidneys, lungs and liver (**Supplementary Figure 5B**). At an early time point (Day 1), high levels of activity were observed in the blood (26.5% ID/g), LNs (25.4% ID/g), the spleen (11.1% ID/g) and kidneys (10.4% ID/g). At a later time point (Day 7), the highest retention of the tracer was measured in LNs (35.9% ID/g), the spleen (17.1% ID/g), liver (16.4% ID/g), blood (10.8% ID/g) and kidneys (10.2% ID/g).

Irrespective of the route of administration, the ^{89}Zr-labeled anti-CD20 mAb reached similar levels in circulation and all major organs except in the spleen and LNs (**Supplementary Figure 5**). In the spleen, the tracer levels following i.v. injection dropped from 24.8% ID/g on Day 1 to 11.4% ID/g at Day 3, before increasing to 24.9% ID/g at Day 7. In contrast, following s.c. injection, the tracer levels in the spleen reached >15% ID/g at Day 3 and remained stable at this level up to Day 7. In the LNs, accumulation of the tracer following i.v. injection showed a peak at Day 3 with >11% ID/g and fell to 8.3% ID/g at Day 7. Following s.c. injection, LNs showed a marked increase in tracer levels, reaching a peak level of >40% ID/g at Day 3 and then dropping to 35.9% ID/g at Day 7.

Supplementary Figure 1. Synthesis and radiolabeling of the anti-CD20-antibody-DFO conjugate. DFO, desferrioxamine; HEPES, (4-(2-hydroxyethyl)-1-piperazineethanesulfonic acid; rpm, rotations per minute; ^{89}Zr, Zirconium-89

Supplementary Figure 2. Study design.
h, hour; PET/CT, positron emission tomography/computed tomography

Supplementary Figure 3. Whole body clearance of the ^{89}Zr-labeled anti-CD20 mAb following s.c. (n=3−6) and i.v. (n=3−8) injection in C57BL/6 healthy mice
Data presented as mean±SD
i.v., intravenous; s.c., subcutaneous; SD, standard deviation; ^{89}Zr, Zirconium-89

Supplementary Figure 4. Comparison of PET/CT in vivo biodistribution (A and B) and in vivo imaging (C and D) of the ^{89}Zr-labeled anti-CD20 mAb following s.c. and i.v. and injection in healthy mice

(A) Biodistribution of the tracer following s.c. injection in healthy mice (n=3−6)
(B) Biodistribution of the tracer following i.v. injection in healthy mice (n=3−8)
(C) In vivo imaging following s.c. injection of the tracer in healthy mice
(D) In vivo imaging following i.v. injection of the tracer in healthy mice

Data presented as mean±SD
% ID/g, percent injected dose per gram; h, hour; i.v., intravenous; PET/CT, positron emission tomography/computed tomography; s.c., subcutaneous; SD, standard deviation; ^{89}Zr, Zirconium-89

Supplementary Figure 5. Comparison of gamma-counter biodistribution of the ^{89}Zr-labeled anti-CD20 antibody following s.c. (A) and i.v. (B) injection in healthy mice

(A) Biodistribution of the tracer following s.c. injection in healthy mice
(B) Biodistribution of the tracer following i.v. injection in healthy mice

Data from the subset of mice (n=3-8); data presented as mean±SD
% ID/g, percentage injected dose per gram; i.v., intravenous; s.c., subcutaneous; SD, standard deviation; ^{89}Zr, Zirconium-89
