# Supplementary figures and images for: Efficient Distribution of a Novel Zirconium-89 Labeled Anti-cd20 Antibody Following Subcutaneous and Intravenous Administration in Control and Experimental Autoimmune Encephalomyelitis-Variant Mice
Source: Front Immunol. 2019 Oct 18;10:2437. doi: 10.3389/fimmu.2019.02437 (PMC6813232; doi:10.3389/fimmu.2019.02437)

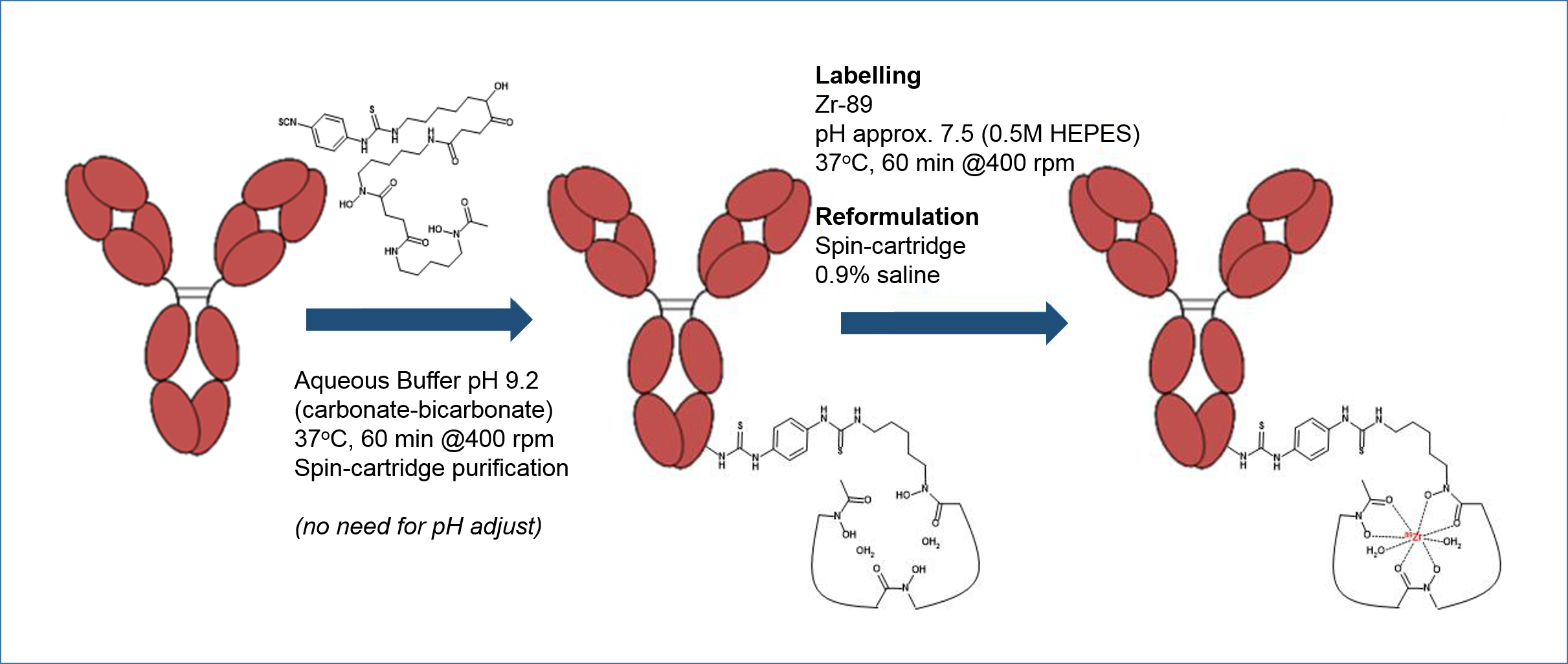

Supplement: Supplementary file 2 [file Image_1.TIF]

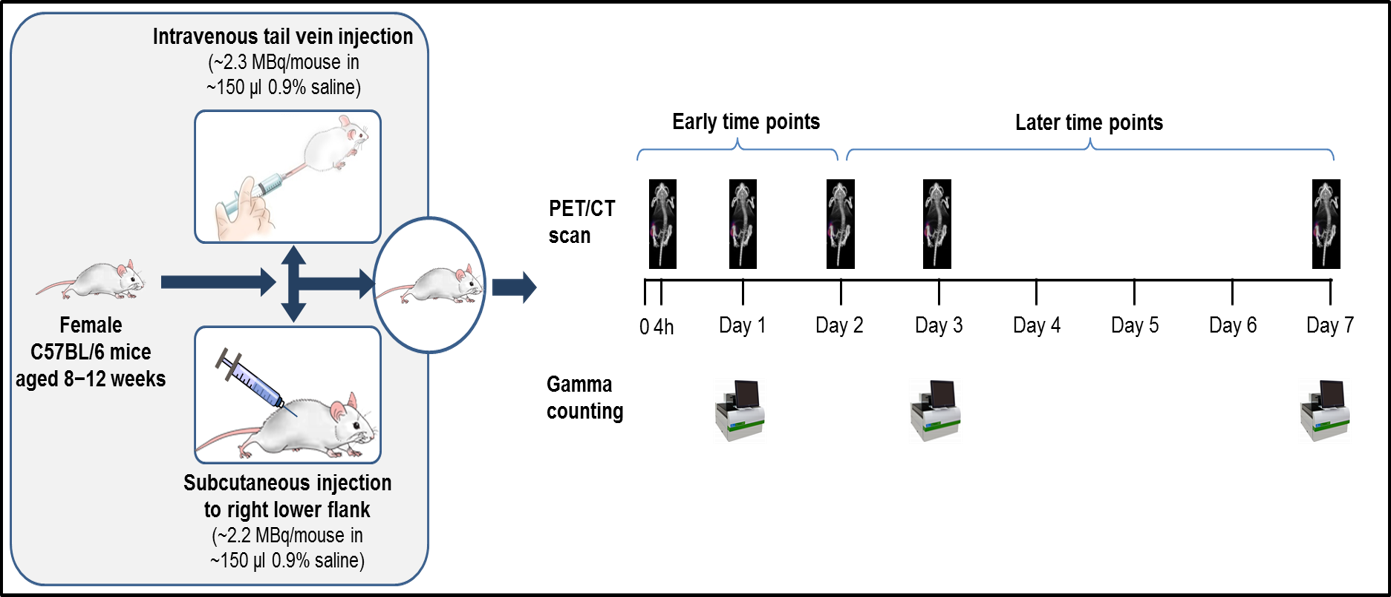

Supplement: Supplementary file 3 [file Image_2.TIF]

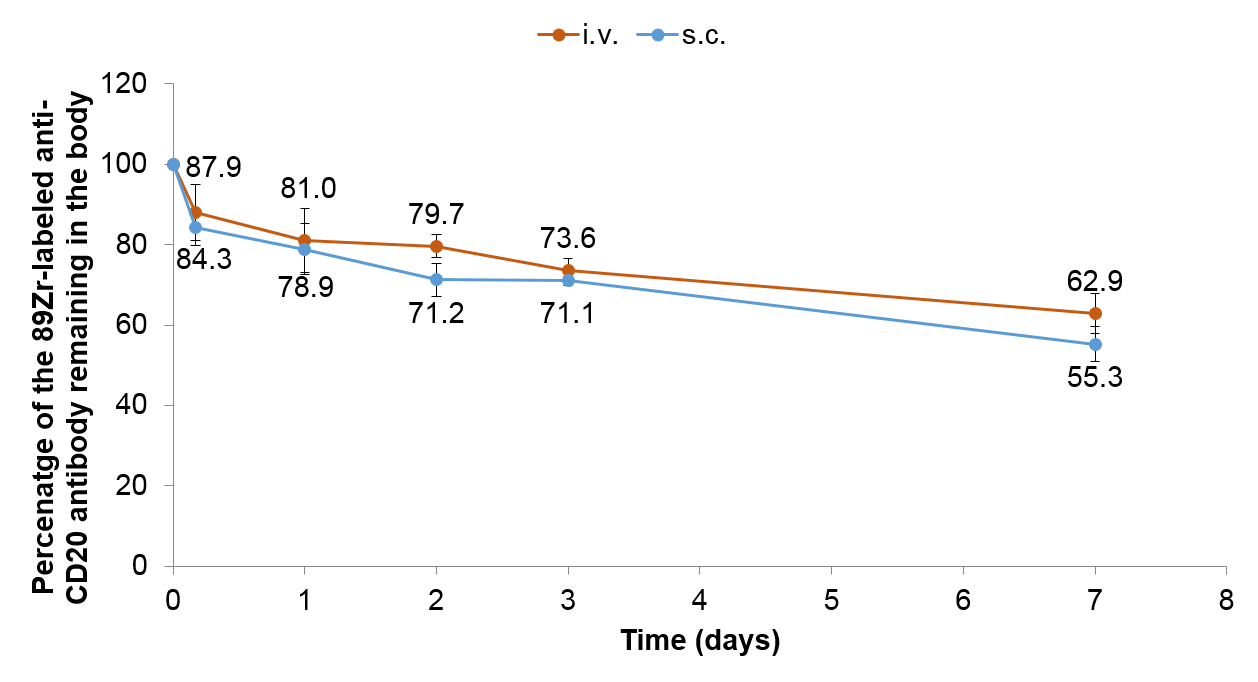

Supplement: Supplementary file 4 [file Image_3.TIF]

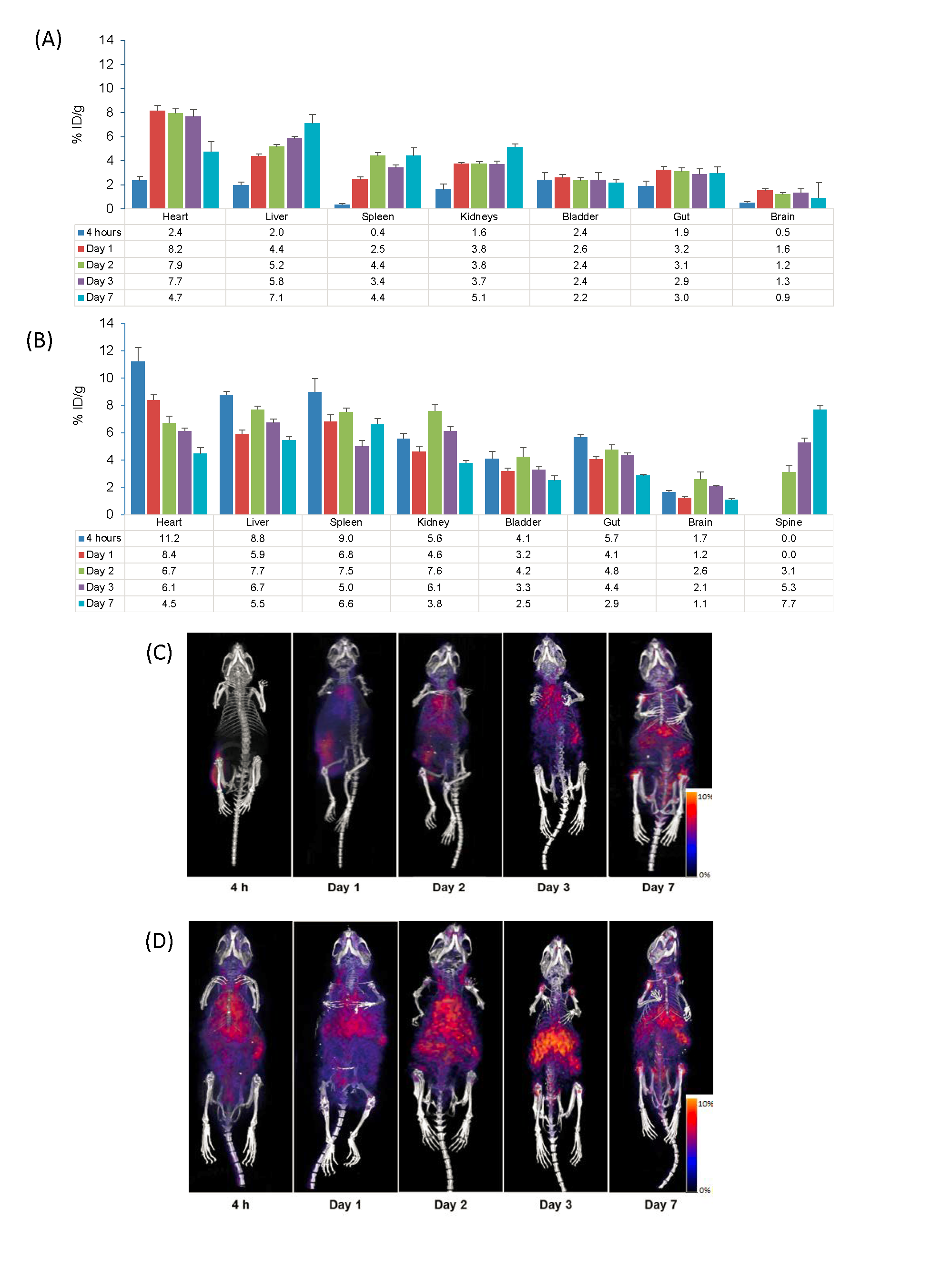

Supplement: Supplementary file 5 [file Image_4.TIFF]

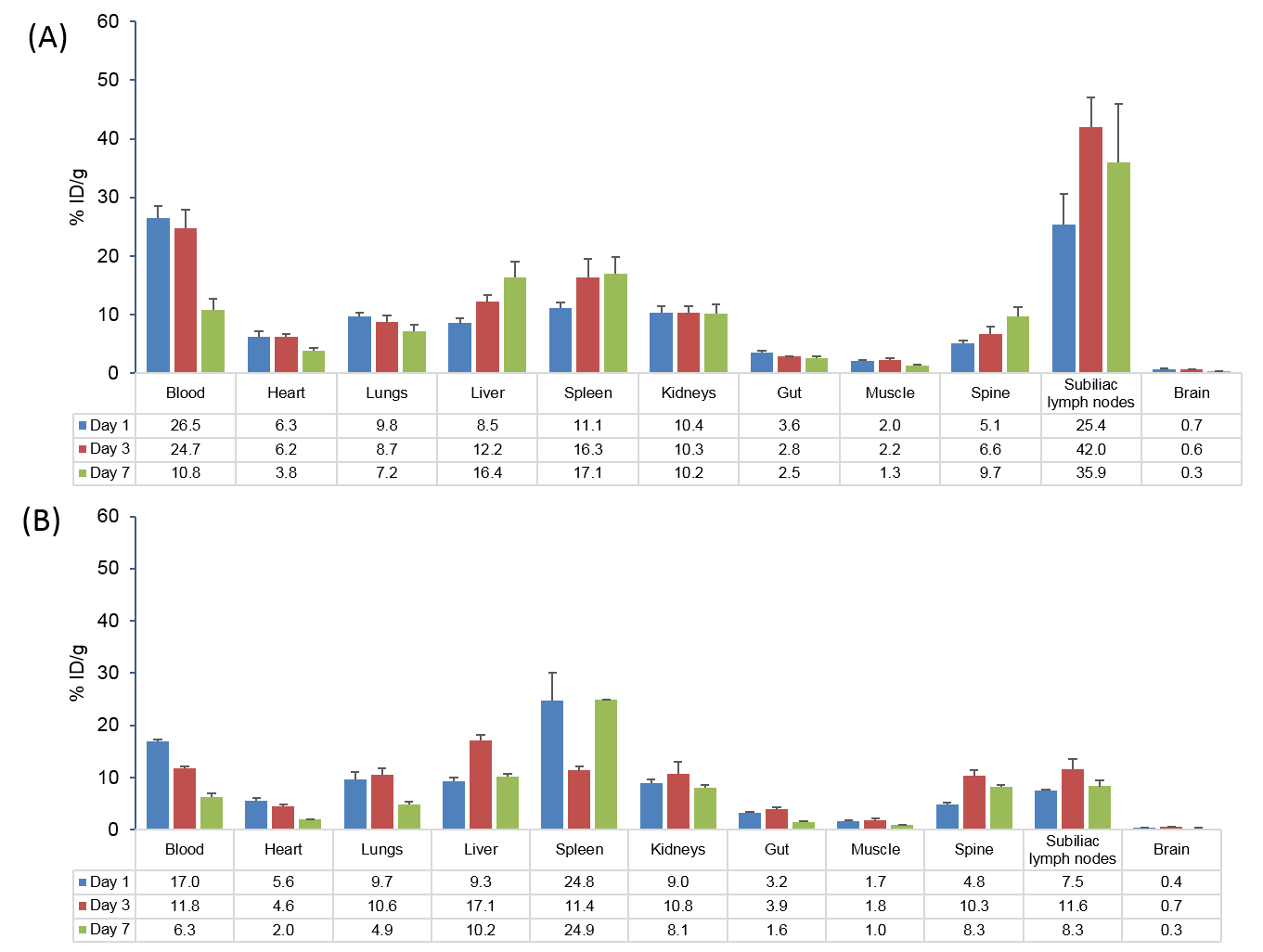

Supplement: Supplementary file 6 [file Image_5.TIF]
